# Au Nanoclusters on Vanadium-Doped ZrO_2_ Nanoparticles for Propylene Oxidation: An Investigation into the Impact of V

**DOI:** 10.3390/ma18051118

**Published:** 2025-03-01

**Authors:** Caixia Qi, Jingzhou Zhang, Xun Sun, Libo Sun, Huijuan Su, Toru Murayama

**Affiliations:** 1 Yantai Key Laboratory of Gold Catalysis and Engineering, Shandong Applied Research Center of Gold Nanotechnology (Au-SDARC), School of Chemistry & Chemical Engineering, Yantai University, Yantai 264005, China; 2 Institute for Catalysis, Hokkaido University, N21W10, Kita-ku, Sapporo 001-0021, Hokkaido, Japan

**Keywords:** gold catalysts, zirconia, vanadium incorporation, propylene epoxidation, catalytic performance

## Abstract

V-doped ZrO_2_ support materials were synthesized through a hydrothermal method, followed by a deposition–precipitation process to load Au clusters using an H_4_AuClO_4_ precursor. This study investigated the impact of vanadium doping on propylene epoxidation over the corresponding Au-supported catalysts. Vanadium incorporation significantly enhanced propylene conversion and promoted acrolein production, leading to reduced propylene oxide selectivity. Propylene epoxidation at higher temperatures accelerated the decomposition of oxygenates into CO_2_. Vanadium addition to ZrO_2_ altered the interactions between Au and V-doped ZrO_2_, thereby modifying the chemical states of Zr, Au, and V and forming surface oxygen vacancies and active oxygen species. These changes defined the catalytic performance of the materials.

## 1. Introduction

Propylene oxide (PO) is a vital chemical intermediate in the production of various chemicals and polymers, including polyurethane, polyester resins, and surfactants [1]. Currently, PO is commercially produced through the chlorohydrin process and several hydroperoxide processes. However, the chlorohydrin process produces a large volume of environmentally toxic chlorinated compounds. Moreover, hydroperoxide processes typically generate significant by-products, leading to high production costs [2]. In contrast, direct gas-phase propylene epoxidation using H_2_ and O_2_ has received attention as an environmentally friendly, simple, and sustainable method.

Since the groundbreaking discovery by Haruta et al. in 1998 [3], significant progress has been made in direct gas-phase propylene epoxidation using H_2_/O_2_ with gold nanoparticle catalysts. The findings revealed that gold nanoparticles (2–5 nm) supported on TiO_2_ were highly effective for PO production, achieving high PO selectivity (>95%) at a propylene conversion of ~1%. This research attracted global interest and led to advancements in Au–Ti catalysts [4]. Au-based catalysts supported on TiO_2_ and various titanosilicates, such as Ti-MCM-41, Ti-MCM-48, Ti-β, TS-2, and TS-1 [5,6,7,8,9,10,11,12,13], have been widely investigated. Except for TS-1, most Au–Ti catalysts exhibit low propylene conversion rates (<2%). However, research on non-Ti-containing supports for propylene epoxidation remains limited, highlighting the need for further investigation.

Zr is located in the same group as Ti on the periodic table and exhibits similar physicochemical properties. Moreover, zirconia (ZrO_2_) has received significant attention as catalyst support owing to its excellent thermal stability, chemical resistance, and redox properties [14,15,16]. Zirconia has been widely used in various oxidation processes, such as volatile organic compounds oxidation [13], CO oxidation [17], and HCHO oxidation [18]. In PO synthesis using ZrO_2_ supports, previous studies have shown that Ag–MoO_3_/ZrO_2_ and Ag–Mo–W/ZrO_2_ catalysts achieved remarkable propylene conversion rates of 1.7% and 15% at 400 °C and 460 °C, respectively [19,20]. In our previous study, Au catalysts supported on ZrO_2_ exhibited excellent PO selectivity. However, the low propylene conversion of these catalysts remains a challenge that needs to be addressed [21].

ZrO_2_ is an effective support with abundant oxygen vacancies that facilitate oxidation reactions. However, ZrO_2_ has certain limitations, such as a limited number of active sites on its surface and a tendency to aggregate at high temperatures. To address these limitations, incorporating V into ZrO_2_ has been proposed. This approach can effectively disperse ZrO_2_ particles and may enhance Au dispersion, potentially increasing the number of active sites [22]. Consequently, the interaction between supported Au and ZrO_2_ will be influenced. Additionally, the incorporation of V species can improve the catalytic performance in propylene epoxidation, thereby affecting the valence state of Zr in ZrO_2_.

This study employed the hydrothermal synthesis method using zirconium nitrate pentahydrate (Zr(NO_3_)_4_•5H_2_O) as the zirconium source and ammonium metavanadate (NH_4_VO_3_) as the vanadium source. Ammonium metavanadate was incorporated into the zirconium source to synthesize the ZrVx support. The impact of different vanadium contents in the Au/ZrVx catalysts on gas-phase propylene epoxidation at various reaction temperatures was investigated. The results revealed that V doping slightly reduced PO selectivity but significantly enhanced propylene conversion. Various characterization techniques were used to assess the effect of V doping on the structure and surface properties of the Au/ZrO_2_ catalyst.

## 2. Materials and Methods

### 2.1. Synthesis of ZrVx Supports

A typical hydrothermal method based on a previously reported procedure [23] was used to synthesize ZrVx (x = 0, 0.01, 0.05, 0.1, and 0.15) supports. The value x represents the molar ratio of V to Zr. The synthesis steps of ZrV_0.01_ are as follows: First, 5.0 g of Zr(NO_3_)_4_•5H_2_O was dissolved in 20 mL of ethanol at 40 °C with stirring. Subsequently, 0.0136 g of NH_4_VO_3_ was added to the mixture and stirred until fully dissolved. Afterward, 0.4 mL of triethanolamine was introduced, and the mixture was vigorously stirred for 30 min to obtain a clear solution (labeled as A). Separately, 0.8 g of CTAB was dissolved in 20 mL of ethanol at room temperature and added dropwise to solution A. After stirring for 1 h, the homogeneous mixture containing Zr–V species was transferred to a Teflon-lined autoclave and heated at 90 °C for 4 h. Once cooled to room temperature, ammonia solution was added to adjust the pH of the reaction mixture to 10–11, and a pale-yellow sol was formed. The sol underwent hydrothermal crystallization at 120 °C for 48 h in a Teflon autoclave. The resulting solid was collected via centrifugation, washed several times with ethanol, and dried overnight at 60 °C. Finally, the dried solid was calcined in static air at 450 °C for 4 h, with the temperature increasing at a rate of 2 °C/min from room temperature to 450 °C.

### 2.2. Deposition of Gold Nanoparticles on ZrVx Supports

A certain amount of HAuCl_4_ solution containing 0.5 wt% Au relative to ZrVx powder was added to a beaker. The pH of the solution was then adjusted to 7 using a 0.1 M KOH solution, followed by the addition of 1 g ZrVx powder. After ultrasonic treatment, the mixture was left undisturbed for 4 h. The mixture was then immersed in ammonia water for 24 h to promote the exchange of chloride ions and washed thoroughly with deionized water until the chloride ions were removed. Finally, the resulting samples were dried overnight at 60 °C and reduced under an H_2_ flow at 300 °C for 1 h. The obtained sample was denoted as Au/ZrVx.

### 2.3. Characterization

Powder X-ray diffraction (XRD) patterns of the samples were obtained using a diffractometer (XRD-6100, Shimadzu Corporation, Kyoto, Japan) with Cu Kɑ radiation (λ = 0.154 nm). Diffraction data were collected in a continuous scan mode over a 2θ range of 5–80° at a scan rate of 5° min^−1^. The BET surface area, pore volume, and pore diameter of the samples were determined through nitrogen adsorption–desorption measurements using a surface area analyzer (Micromeritics, Norcross, GA, USA, ASAP2020HD). Before measurements, each sample was degassed at 200 °C for 3 h. Raman spectra were recorded using a Senterra spectrometer (Bruker, Billerica, MA, USA) with a 532 nm laser source and a spectral range of 40–4000 cm^−1^. The gold content in Au/ZrVx was measured via inductively coupled plasma optical emission spectroscopy (ICP-OES) (Agilent 5110, Agilent Technologies, Santa Clara, CA, USA). The acidity of the samples was analyzed through temperature-programmed desorption of ammonia (NH_3_-TPD) using a TP-5080 instrument (Xianquan, Xi’an, China). A 0.1 g catalyst sample was pretreated at 300 °C in a helium flow (30 mL min^−1^) for 30 min. The sample was exposed to ammonia gas for 30 min at room temperature to enable NH_3_ adsorption and then purged with He for 30 min. The temperature was gradually increased from 50 °C to 750 °C at a rate of 10 °C/min, and NH_3_ desorption was monitored using a thermal conductivity detector (TCD) detector. The oxidation states of Zr, V, Au, and O were determined via X-ray photoelectron spectroscopy (XPS) using a Perkin-Elmer analyzer (Waltham, MA, USA) with AlKa X-ray radiation (1486.6 eV). The C1s peak at 284.8 eV served as the reference for binding energy calibration.

### 2.4. Propylene Oxidation

For propylene oxidation, 0.15 g of the prepared catalyst was placed in a vertical fixed-bed U-shaped quartz tubular reactor (8.0 mm inner diameter) equipped with a thermocouple. The catalyst was heated under a continuous flow of a reaction gas mixture (C_3_H_6_/H_2_/O_2_/Ar = 10/10/10/70 vol%) at a rate of 4000 mL∙gcat^−1^∙h^−1^ from room temperature to 200 °C. An online analysis of reactants and products was performed using two gas chromatographs (Agilent 7820A). A flame ionization detector with an FFAP capillary column and a TCD with Porapak Q and 5A columns were used to detect C_2–3_ oxygenates and inorganic gas products, respectively. The carbon balance in all experiments was consistently close to 100%.

To ensure data accuracy, each temperature point was tested twice, and two identical samples were analyzed repeatedly. Before the catalytic data were collected, the catalyst was stabilized at the test temperature for 25 min. The experimental system exhibited high reproducibility, with retention time variations below 0.0008 min and peak area deviations within 1% RSD.

## 3. Results and Discussion

**Figure 1:**
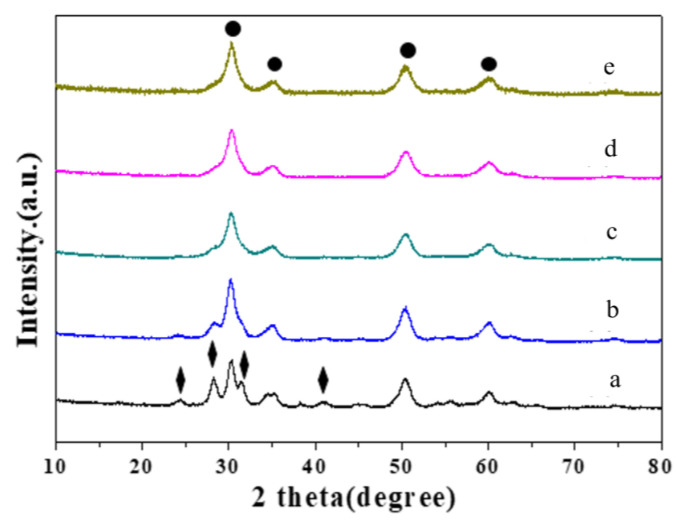
XRD patterns of 0.5 wt% Au/ZrVx catalysts: (a) 0.5 wt% Au/ZrO_2_, (b) 0.5 wt% Au/ZrV_0.01_, (c) 0.5 wt% Au/ZrV_0.05_, (d) 0.5 wt% Au/ZrV_0.1_, and (e) 0.5 wt% Au/ZrV_0.15_. (●: tetragonal ZrO_2_, ◆: monoclinic ZrO_2_).

Figure 1 shows the XRD patterns of the obtained 0.5 wt% Au/ZrO_2_ and 0.5 wt% Au/ZrVx catalysts (x = 0.01, 0.05, 0.1, and 0.15). The XRD pattern of 0.5 wt% Au/ZrO_2_ revealed diffraction peaks at 2θ = 30, 35, 51, and 60°, corresponding to tetragonal ZrO_2_ (JCPDS card No. 79-1976). Additionally, diffraction peaks at 2θ = 24.4, 28.1, 31.5, and 41.4° were assigned to monoclinic ZrO_2_ (JCPDS card No. 78-0047). As the V/Zr molar ratio increased from 0 to 0.15, the intensity of the monoclinic ZrO_2_ peaks decreased. Notably, 0.5 wt% Au/ZrV_0.1_ and 0.5 wt% Au/ZrV_0.15_ exhibited only the tetragonal phase. This suggests that the incorporation of an appropriate amount of V promoted the crystallization of the tetragonal phase over the monoclinic phase [22]. No diffraction peaks attributed to the Au phase (at around 2θ = 38.2, 44.4, and 64.6°) were detected owing to the high dispersion of Au species or low Au loading [24]. Moreover, at a high vanadium doping level of V/Zr = 0.15 (mol/mol), no diffraction peaks corresponding to crystalline V_2_O_5_ or other vanadium oxides were detected. This was in contrast to previously reported supported catalysts [25,26]. This indicates that vanadium oxide is highly dispersed in the bulk and/or on the zirconia surface.

**Figure 2:**
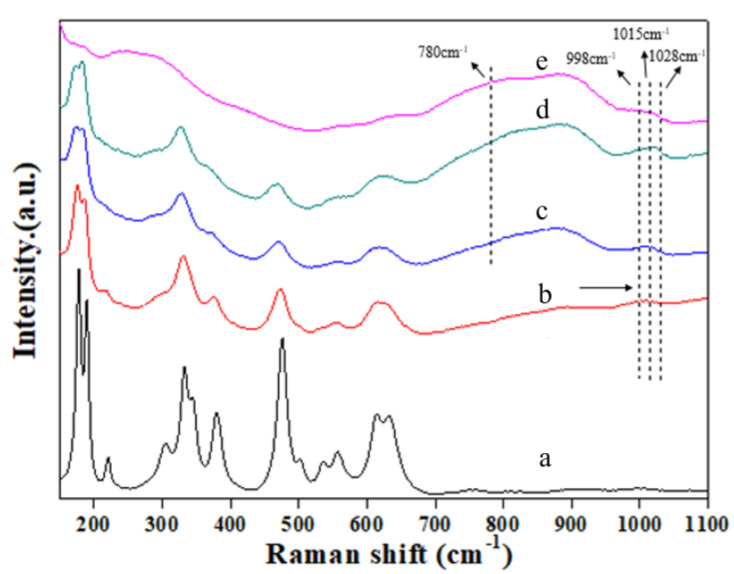
Raman spectra of 0.5 wt% Au/ZrVx catalysts with different V doping levels. (a) 0.5 wt% Au/ZrO2, (b) 0.5 wt% Au/ZrV_0.01_, (c) 0.5 wt% Au/ZrV_0.05_, (d) 0.5 wt% Au/ZrV_0.1_, and (e) 0.5 wt% Au/ZrV_0.15_.

Raman scattering measurements were conducted to characterize the vanadium species in the obtained samples. Figure 2 shows the Raman spectra of 0.5 wt% Au/ZrVx catalysts deposited as a film on Si. The Raman spectra of 0.5 wt% Au/ZrO_2_ catalysts exhibited bands at 179, 334, 381, 476, 615, and 613 cm^−1^, characteristic of the monoclinic phase. Bands at 315 and 643 cm^−1^ corresponded to the tetragonal phase of zirconia [27,28,29,30,31]. However, with the increasing vanadium content, all the bands in ZrO_2_ became broader and weakened in intensity, eventually disappearing at the highest vanadium doping level (V/Zr = 0.15 mol/mol). These changes were consistent with the Raman spectra of supported vanadium oxides [32], indicating that vanadium doping altered or distorted the surface Zr–O bonds. The Raman bands at 780 and 998 cm^−1^ were assigned to the V–O–V and V=O stretching modes, respectively, corresponding to polyvanadate species (ZrV_2_O_7_) and V_2_O_5_. Additionally, a Raman band at 1028 cm^−1^ was observed in the 0.5 wt% Au/ZrV0.1 and 0.5 wt% Au/ZrV0.15 samples, corresponding to the V=O stretching mode of isolated monovanadate species. In 0.5 wt% Au/ZrV_0.05_, this band shifted to 1015 cm^−1^ [32,33,34], likely owing to low surface vanadium coverage, similar to that observed in the V/TiO_2_ system [33].

**Figure 3:**
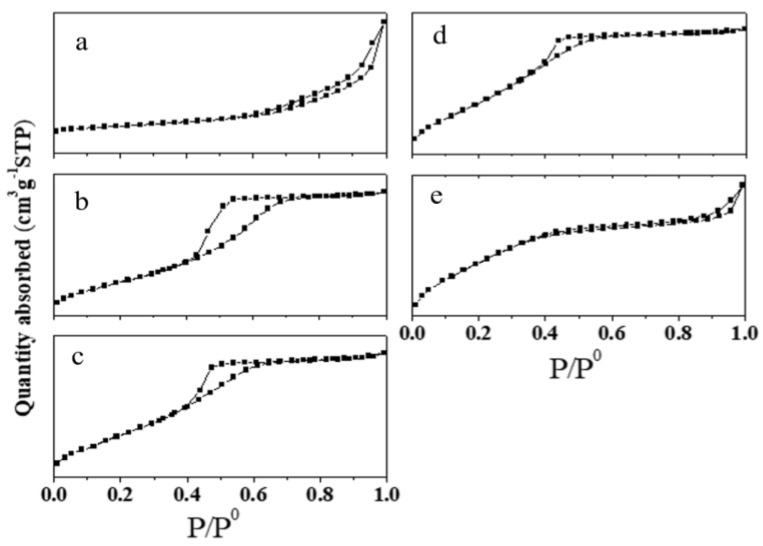
N_2_ adsorption–desorption isotherms of 0.5 wt% Au/ZrVx catalysts. (**a**) 0.5 wt% Au/ZrO_2_, (**b**) 0.5 wt% Au/ZrV_0.01_, (**c**) 0.5 wt% Au/ZrV_0.05_, (**d**) 0.5 wt% Au/ZrV_0.1_, and (**e**) 0.5 wt% Au/ZrV_0.15_.

The N_2_ adsorption–desorption isotherms of all the prepared samples (Figure 3) exhibited type IV characteristics, indicating mesoporous structures. The 0.5 wt% Au/ZrV_0.01_, 0.5 wt% Au/ZrV_0.05_, and 0.5 wt% Au/ZrV_0.1_ samples displayed H1 hysteresis loops, suggesting a homogeneous mesopore distribution. Conversely, the 0.5 wt% Au/ZrO_2_ and 0.5 wt% Au/ZrV_0.15_ catalysts exhibited elongated hysteresis loops, corresponding to H3 and H4 types, which were associated with slit-shaped pores. This indicates the presence of some cumulative macropores. The specific surface areas and pore diameters of the obtained catalysts were calculated through the BET method and are summarized in Table 1. As the vanadium doping increased from 0.5 wt% Au/ZrO_2_ to 0.5 wt% Au/ZrV_0.15_, the specific surface area increased, while the pore diameter decreased. This trend may be attributed to the smaller particle size of the support, as discussed below.

**Figure 4:**
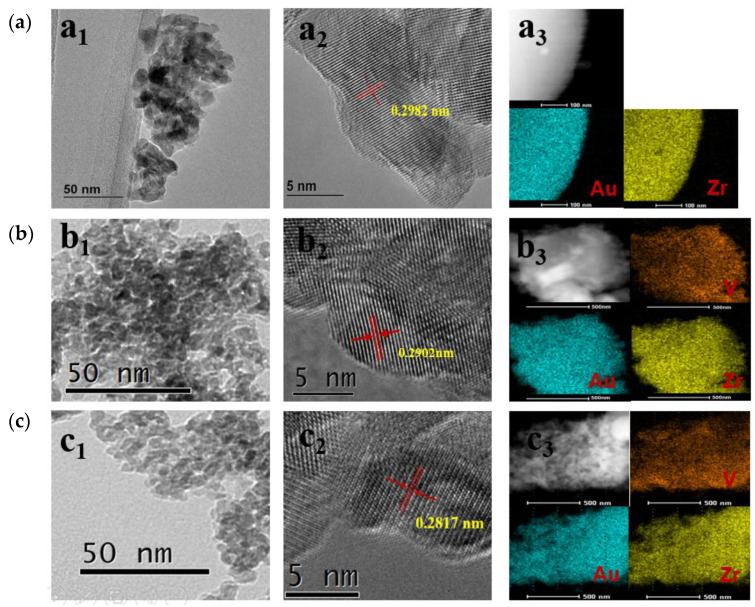
TEM images (**a1**,**b1**,**c1**), HRTEM electron diffraction patterns (**a2**,**b2**,**c2**), and HAADF images with corresponding elemental mappings (**a3**,**b3**,**c3**) of the samples: (**a**) 0.5 wt% Au/ZrO_2_, (**b**) 0.5 wt% Au/ZrV_0.01_, and (**c**) 0.5 wt% Au/ZrV_0.15_.

Figure 4 shows the TEM images of 0.5 wt% Au/ZrO_2_, 0.5 wt% Au/ZrV_0.05_, and 0.5 wt% Au/ZrV_0.15_ samples. The average particle size of the support oxide was calculated from TEM measurements and is summarized in Table 1. With the addition of vanadium, the particle size decreased from 14.8 nm in 0.5 wt% Au/ZrO_2_ to 6.9 nm in 0.5 wt% Au/ZrV_0.15_. This trend aligned with the crystallite size calculated using Scherrer’s equation from XRD analysis at 2θ = 30, 35, 51, and 60°. The crystallite size decreased from 15.5 to 7.8 nm with increasing vanadium content. This observation was consistent with previous literature findings [21,35] and suggests that the addition of the second metal increased the specific surface area of the ZrO_2_ support. Notably, no visible Au particles were observed in the HAADF–STEM images of 0.5 wt% Au/ZrO_2_, 0.5 wt% Au/ZrV_0.05_, and 0.5 wt% Au/ZrV_0.15_. However, elemental mapping confirmed the uniform dispersion of both Au clusters and V elements on the ZrVx support. Additionally, the crystal lattice spacing measured in the HRTEM–STEM images (Figure 4(a2,b2,c2)) were 0.2982, 0.2902, and 0.2817 nm, indicating a decreasing trend with increasing vanadium content. This suggests that V^5+^ (or V^4+^), with a smaller ionic radius than Zr^4+^ (0.072 nm), partially replaced Zr in the lattice, leading to a slight shrinkage in lattice spacing [36]. These findings indicate that vanadium doping effectively prevented ZrO_2_ particle aggregation and reduced its particle size. Because ZrO_2_ particles naturally tend to aggregate, vanadium doping can mitigate this tendency, thereby increasing the specific surface area of the ZrO_2_ support.

ICP–OES measurements were conducted to evaluate the effect of different vanadium contents on gold loading. The actual Au loading was determined, and the results (Table 1) indicated that vanadium content had no significant effect on Au loading.

**Figure 5:**
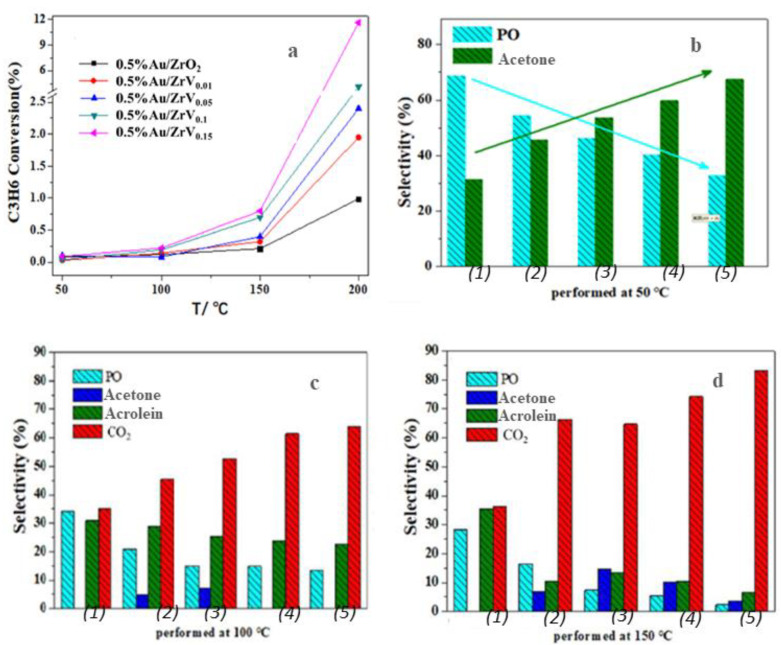
Catalytic performance of 0.5 wt% Au/ZrVx in propylene epoxidation. (**a**) Propylene conversion, (**b**) product selectivity at 50 °C, (**c**) product selectivity at 100 °C, and (**d**) product selectivity at 150 °C. Catalysts: (1) 0.5 wt% Au/ZrO_2_, (2) 0.5 wt% Au/ZrV_0.01_, (3) 0.5 wt% Au/ZrV_0.05_, (4) 0.5 wt% Au/ZrV_0.1_, and (5) 0.5 wt% Au/ZrV_0.15_.

To investigate the effect of different vanadium-to-zirconium molar ratios on the catalytic performance of 0.5% Au/ZrVx catalysts in the propylene epoxidation reaction, the temperature was gradually increased from room temperature at a rate of 2 °C/min, with a space velocity of 4000 mL·gcat^−1^·h^−1^. The reaction was stabilized at 50 °C at 25 min intervals, and online sampling for product analysis was conducted until the temperature reached 300 °C. Each temperature point was sampled and analyzed twice, with online sampling performed every 25 min. Before reaction testing, all the catalysts were pretreated in a hydrogen atmosphere at 300 °C for 1 h. The results revealed that nearly all the catalysts remained inactive at room temperature. However, at reaction temperatures of ≥200 °C, PO selectivity was nearly zero. As the reaction temperature increased from 50 °C to 200 °C, the propylene conversion rate gradually increased across all the catalysts (Figure 5). The distribution of reaction products varied with increasing temperature and vanadium content. Notably, no PO activity was detected using pristine ZrVx supports without Au, indicating the vital role of Au in PO synthesis.

With the increasing V content in the support, propylene conversion rates increased across all the reaction temperatures (Figure 5a). For example, the propylene conversion rates increased from 0.08% (0.5 wt% Au/ZrO_2_) to 0.99% (0.5 wt% Au/ZrV_0.15_) at 50 °C and from 0.09% (0.5 wt% Au/ZrO_2_) to 11.6% (0.5 wt% Au/ZrV0.15) at 200 °C, respectively. However, unlike the conversion trend, PO selectivity decreased with the increasing V/Zr molar ratio. At a lower reaction temperature of 50 °C, only PO and acrolein were produced (Figure 5b). With the increasing vanadium loading, the PO selectivity gradually decreased from 68.7 to 32.6, while acrolein selectivity increased from 31.3% to 67.4%. This indicates that vanadium facilitated acrolein formation.

At a reaction temperature of 100 °C (Figure 5c), the product distribution included PO, acrolein, CO_2_, and a small amount of acetone. With increasing vanadium content, PO selectivity significantly decreased from 34.1% to 14.3%, while acrolein selectivity decreased from 30.8% to 22.6%. Conversely, CO_2_ selectivity significantly increased from 35.1% to 63.9%. At 150 °C (Figure 5d), the decrease in PO selectivity and the increase in CO_2_ selectivity became more pronounced.

## 4. Discussion

The improvement in catalytic activity with vanadium addition can be attributed to the increased specific surface area of the ZrVx support, which improves Au dispersion and provides more active sites. Alternatively, if Au dispersion remains similar, variations in the electronic interactions between the ZrVx support and Au due to the V content may influence the catalytic performance of Au as the active site.

**Figure 6:**
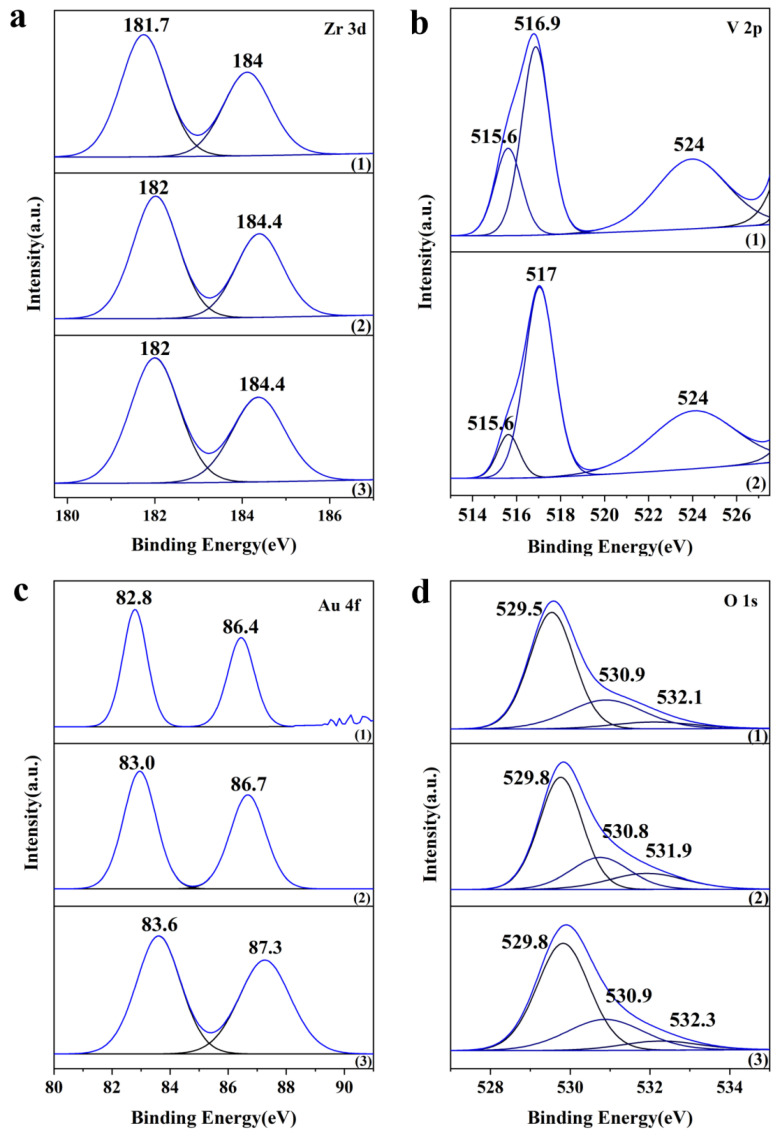
XPS spectra of 0.5 wt% Au/ZrVx catalysts. (**a**) Zr 3d, (**b**) V 2p, (**c**) Au 4f, and (**d**) O1s. Catalysts: (1) 0.5 wt% Au/ZrO_2_, (2) 0.5 wt% Au/ZrV_0.05_, and (3) 0.5 wt% Au/ZrV_0.15_.

The chemical state of surface species significantly influenced catalyst activity. The Zr 3d spectra of all the catalysts (Figure 6a) exhibit spin–orbit doublets with binding energies—lower than the typical Zr 3d5/2 value of 182.2 eV. This confirms the partial reduction of surface Zr^4+^ to Zr^3+^ species [37]. The XPS spectra of V-containing samples (0.5 wt% Au/ZrV_0.05_ and 0.5 wt% Au/ZrV_0.15_) (Figure 6b) exhibited a peak at 517.1 eV and a shoulder peak at 515.6 eV. This suggests a partial reduction of V^5+^ to V^4+^ species [38], with the proportion of V^4+^ increasing as the V content increased. Figure 6c shows a characteristic peak at 83.6 eV for the Au 4f7/2 binding energy in the 0.5 wt% Au/ZrO_2_ sample, indicating the presence of metallic gold [39]. The XPS spectrum of Au 4f7/2 in the V-doped samples (0.5 wt% Au/ZrV_0.05_ and 0.5 wt% Au/ZrV_0.15_) displayed binding energies at 83.0 and 82.9 eV, respectively. This shift to lower binding energies indicated the reduction of the Au precursor to its metallic state owing to V doping, which donates excess electrons. The production of Zr^3+^, V^4+^, and Au^0^ with excess electrons can be attributed to oxygen vacancy generation during H_2_ pretreatment, which promotes acrolein formation [21]. Particularly, H_2_ reacted with surface O^2−^ to form water molecules and create oxygen vacancies. These vacancies released electrons, which were captured by metallic Au, Zr^4+^, and V^5+^ on the catalyst surface.

The two 0.5%Au/ZrVx catalysts exhibited a higher amount of surface-activated oxygen species (Figure 6d). This indicates that an appropriate level of V doping in the ZrO_2_ structure can accelerate electron transfer to the antibonding orbitals of O_2_ molecules, thereby promoting oxygen activation and O^2−^ species formation.

Metallic Au species facilitated the selective generation of PO through propylene epoxidation [40], while the acid sites of the support promoted subsequent PO oxidation, leading to enhanced propylene oxidation and reduced PO selectivity [22]. The catalytic activity results, the increasing acidity amount with V content (Table 1), and the trends in the binding energies of V, Zr, and Au indicated that V doping affected the interactions between Au, Zr, and V. This interaction influenced the content of metallic Au, surface oxygen vacancies, active oxygen species, and acidity. These factors contributed to the catalytic performance of the system. Currently, it is challenging to quantitatively correlate the amount of V^4+^ or V^5+^ species with PO yield, despite the increase in propylene conversion with higher vanadium doping in the ZrO_2_ substrate. Further studies can focus on modifying the pretreatment atmosphere of the catalyst precursor before catalytic testing. Additionally, further reaction tests using feed with low H_2_ content or without hydrogen are anticipated, consistent with our recent study on V-doped TS-1-supported Au catalysts [41].

## 5. Conclusions

A series of 0.5 wt% Au/ZrVx catalysts were prepared and evaluated for direct propylene oxidation in the presence of H_2_ and O_2_. The effect of vanadium doping on the physicochemical properties and catalytic performances of the catalysts was investigated. The results revealed that V doping significantly reduced the ZrO_2_ particle size, thereby preventing particle aggregation. Both Au and vanadium species were highly dispersed on the catalysts. The highly dispersed catalysts achieved higher propylene conversion, but PO selectivity decreased owing to increased acrolein production. The increase in propylene conversion and the decrease in PO selectivity can be attributed to the strong interaction between Au and the V-doped ZrO_2_ support, with optimal vanadium content. The production of Zr^3+^, V^4+^, and Au^0^ with excess electrons can be attributed to oxygen vacancies formed during H_2_ pretreatment, which promoted acrolein production. Our findings provide insights into Ti-free, V-doped Au-based catalysts and valuable guidance for developing high-efficiency catalysts to enhance catalytic activities in direct propylene epoxidation.

## Figures and Tables

**Table 1:** Properties of 0.5 wt% Au/ZrVx catalysts with different V/Zr ratios.

Catalysts	S_BET_ (m^2^ g^−1^)	Average Particle Size (nm) ^a^	Crystal Size (nm) ^b^	Pore Vol. (cm^3^ g^−1^) ^c^	Pore Size (nm) ^d^	Au Loading (wt%)	Acid Amount (mmol g^−1^) ^e^
0.5 wt% Au/ZrO_2_	85	14.8	15.5	0.32	15.2	0.33	0.116
0.5 wt% Au/ZrV_0.01_	101	-	10.3	0.17	4.6	0.27	0.166
0.5 wt% Au/ZrV_0.05_	197	9.4	9.2	0.18	3.7	0.23	0.292
0.5 wt% Au/ZrV_0.1_	193	-	8.3	0.15	3.1	0.27	0.362
0.5 wt% Au/ZrV_0.15_	249	6.9	7.8	0.19	3.1	0.22	0.452

^a^ Determined through TEM observation. ^b^ Calculated from XRD analysis using the Scherrer equation. ^c^ Derived from the volume adsorbed at P/P_0_ = 0.99. ^d^ Calculated using the BJH model (desorption). ^e^ Determined via the NH_3_-TPD method.

## Data Availability

The original contributions presented in this study are included in the article. Further inquiries can be directed to the corresponding author.
